# Sizing Up Objects: The Effect of Diminutive Forms on Positive Mood, Value, and Size Judgments

**DOI:** 10.3389/fpsyg.2016.01452

**Published:** 2016-09-23

**Authors:** Michał Parzuchowski, Konrad Bocian, Pascal Gygax

**Affiliations:** ^1^Sopot Social Cognition Lab, SWPS University of Social Sciences and HumanitiesSopot, Poland; ^2^Department of Psychology, University of FribourgFribourg, Switzerland

**Keywords:** language, diminutive forms, judgments, size, satisfaction, value, cognition

## Abstract

Language (e.g., structure, morphology, and wording) can direct our attention toward the specific properties of an object, in turn influencing the mental representation of that same object. In this paper, we examined this idea by focusing on a particular linguistic form of *diminution* used in many languages (e.g., in Polish, Spanish, and Portuguese) to refer to an object as being “smaller.” Interestingly, although objects are usually considered “better” when they are bigger in size, objects described with linguistic diminution can also refer to those that are emotionally positive. Across three experiments conducted in Polish, we examined this lexical ambiguity in terms of mood (Experiment 1), subjective quality and monetary value (Experiment 2), and choice selection (Experiment 3). Overall, we found that people evaluate objects differently depending on the linguistic form (i.e., with or without diminution) with which they are described, and that it was related to the perceptual representation of these objects, and not their affective status. Objects described with diminution are evaluated as less satisfying and of lesser value and this effect is attributed to the way participants represent the objects (i.e., encoded and memorized). The generalizability of these effects is discussed.

## Introduction

Imagine being invited to a birthday party of one of your friends. You are very excited, because you found the one gift that you know will please him: a mug with the picture of a cat in a hat on it. When your friend gets your present he says: “Oh! What a nice *little* present!” In English, there is some ambiguity in his use of the term *little*. He could be possibly referring to either the physical size (smallness) of the object (i.e., “*This is a little gift*”) or the affective status – in terms of how nice it is – of it (i.e., “*Such a lovely, little gift*”). In other languages (e.g., Spanish or Polish), the ambiguity in valence is much stronger, inasmuch as both meanings are very frequent when using language cues, such as diminutive forms, referring to size (Haman, 2003).

Diminutive forms of spoken language are exceptionally common among users of many Romance (e.g., Spanish, Portuguese, or Italian), Slavic (e.g., Polish, Czech, or Russian) and Germanic (e.g., Dutch or German) languages. Spanish and Polish are particularly rich in their use of diminutive suffixes, as almost any noun, adjective, or adverb can take a diminutive form [e.g., *centav-ito* or *pieniaaaa-żki* (a small amount of money = *money*_with_ _diminutive_) instead of *centa-vo* or *pieniaaaa-dze* (money)]. As mentioned earlier, the diminutive form can signal either (a) the relative small size of an object or (b) the speaker’s familiarity and affection toward the same object (1). This suggests a certain level of ambiguity, in that the use of the diminutive form is not limited only to describing objects of smaller size or quantity. In fact, diminutive forms are often used when describing precious and beloved objects. For example, the diminutive form of the noun “*coin”* [e.g., *złotóweczka* (a coin_with diminutive_) instead of *złotówka* (a coin)] can either suggest that it is precious, or simply that it is smaller. In terms of the mental processes involved – and their resulting mental representations–, determining which meaning is dominant has yet to be empirically tested. At the very base of this issue is the fact that the use of the diminutive forms does not necessarily mean that the object *is* physically smaller.

Also, importantly, whilst in some languages (e.g., English), linguistic diminution is rather informal and used mostly in the context of child directed speech (*doggie* vs. *dog*; *horsey* vs. *horse*; Burnham et al., 2002) or nicknames between friends (*Maggie* vs. *Margaret*; Bombar and Littig, 1996), in Polish or Spanish, diminutives are very common and are frequently used in spoken language, even in formal settings (e.g., *ratka* vs. *rata*–a mortgage fee; *momento* vs. *momentito–* a moment; Mendoza, 2005; Królak and Rudnicka, 2006).

We argue that this unmarked use of diminutive nouns when describing objects constrasts the opposite meanings of “*very small*” vs. “*really nice.*” However, we essentially argue that for many objects, especially those sensitive to the heuristic *bigger is better*, the use of diminutive forms will predominantly generate smaller perceptual representations. If the prevailing interpretation of the diminutive form was to be of a physical nature (i.e., the object is represented as being smaller), a number of corollary processes may come into effect. Several studies have actually found that the physical size of an object may be used as a heuristic to process that object (Josephs et al., 1994, 1996; Silvera et al., 2002; Meier et al., 2008). Silvera et al. (2002), for example, showed that people, aesthetically speaking, prefer bigger (abstract) objects. This suggests that the size of an object acts as a multi-purpose heuristic (Bromgard et al., 2013) and that the *bigger is better* rule is predominantly used when evaluating objects. Some authors have argued this predominance to be grounded in evolutionary processes (e.g., Leaky, 1971; Campbell, 1976), as it can also be observed in the animal kingdom: social benefits are reserved for those that can effectively increase their body size (e.g., baboons, peacocks, cats, or porcupines; Alcock, 1984). It might have been the case that at one point in the evolutionary process, considering bigger things as better was a proficient way to consider the environment. Although this is clearly not the case anymore, at least for some objects that strive for miniaturization (e.g., electronics, medical instruments), there might still be mental residues of these evolutionary processes.

Regardless of these evolutionary grounds, the possibility that language, through the use of diminutive forms, can alter the perceptual representation of an object’s size (and possibly subsequent processing) is reminiscent of the *thinking for speaking* principle put forward by Slobin (2003). Because language provides only a limited set of options for encoding certain properties of objects, some linguistic forms may be bound to certain characteristics of these objects. In turn, when encountering these specific forms, our attention may be inevitably and excessively directed toward these underlying characteristics. There is ample evidence that properties of language can potentially provide us with readily available cues on which to base our evaluations of objects (e.g., reading comprehension; Garner, 1987; framing effects; Kahneman and Tversky, 1973, 1996; priming effects; Neely, 1977; Higgins et al., 1985; Semin, 2008; Boroditsky, 2009), or even our evaluation of people in terms of gender (e.g., Sato et al., 2013). In other words, language shapes our mental representations, regardless of comprehenders’ processing dispositions.

However, we do not argue that the use of the diminutive form will influence *perception per se*, (i.e., visual input information that participants actually perceive are exactly the same regardless of the description), but more the way the objects are represented, that is, encoded and memorized. Some authors, such as Lupyan (2012, 2015) and Lupyan and Ward (2013), have argued that lower-level operations could be influenced by higher-level cognitive contexts such as language (Goldstone, 1995; Levin and Banaji, 2006; Lupyan and Spivey, 2008; Lupyan et al., 2010), yet we believe that these authors may have overestimated the extent to which top-down processes could influence perception, as rightly put forward recently by Firestone and Scholl (2014, 2015). Firestone and Scholl (2015) argued that it is unlikely that cognition affects the way we *see* the world. It may affect other higher-level processes, such as encoding or memory, but not perception *per se*. So, for example, they argue that the fact that wearing heavy backpacks makes hills look steeper (Bhalla and Proffitt, 1999) is only an artifact of the experimental demand. If participants are given a rationale for wearing a heavy backpack (e.g., carying heavy monitoring equipment, as in Durgin et al., 2009), the effect disappears. As interesting as these effects are, we do not wish to address this controversial debate here, as it goes beyond the scope of this paper.

We mainly wish to argue that the linguistic forms under investigation in this paper will have an effect on the way the perceived objects will be mentally processed, that is, encoded and memorized. What we do not know, though, is the way our cognitive system may deal with the ambiguity resulting from the use of diminutive forms (i.e., “*very small*” vs. “*really nice*”). Accessing perceivers’ mental representations when processing these forms should enlighten us in this matter.

### Bigger Is Better, Smaller Is Less Positive

The notion that people often make judgments under uncertainty based on a small number of simplifying strategies (i.e., heuristics), rather than on all available information, or extensive algorithmic processing, is undisputed (Kahneman and Tversky, 1973, 1996; Tversky and Kahneman, 1974, 1986; Kahneman, 2000; Gilovich et al., 2002; Kahneman, 2003). For example, when making qualitative judgments about objects, people often use simple and apparent contextual cues, such as the objects’ physical size, when indicating *aesthetic preferences* (Josephs et al., 1994, 1996; Silvera et al., 2002).

In fact, perceptual properties, such as physical size, are often part of the mental representations that are partially based on the same system as conceptual knowledge (Barsalou, 1999; Shen et al., 2016). Shen et al. (2016), for example, showed that when presenting a pair of words referring to objects of different sizes (e.g., grape – watermelon), participants were more accurate (and faster, in Experiment 3b) when judging if one of the objects was smaller or bigger than the other if the object was written in a congruent font size (i.e., bigger font for a bigger object). Importantly, the effect was only present if participants were forced to semantically process the words. In our case, and following the *bigger is better* principle, if the diminutive form is processed as a cue for the physical size of an object (i.e., it is smaller), we believe that the mental (conceptual and/or semantic) representations of that object will include the notion that it is of lesser value.

### The Present Paper

Thus, in the current paper, we examine three issues. First, we explore whether a diminutive form (in Polish), even if its interpretation can be ambiguous, is an exploited cue for the physical size of a described object. If it is, regardless of the pragmatic intentions associated with the use of the diminutive form, it may be perceived as something rather negative (i.e., smaller is less positive than bigger). Second, we examine whether the resulting perceptual mental representation has an impact on (a) the satisfaction of a person receiving an object described with the diminutive form, (b) the subjective quality of that object and (c) its pecuniary value. Third, we investigated whether diminutive labels of objects, through their constructed mental representations, could influence selection processes of exemplars differing in their actual size.

Consequently, the goal of the present paper was twofold: to demonstrate the influence of a linguistic cue (a) on the perceptual representation of an object and consequently (b) on various qualitative judgments of that object. More precisely, we hypothesize that the ambiguity associated with the use of a diminutive form is resolved taking *size* as the predominant criterion. On that account, people are less satisfied when receiving a single gift described with the diminutive form than when it is described normally (Experiment 1). We also hypothesize that pieces of clothing are judged to be of lesser value when described using the diminutive form (Experiment 2), and, finally, in Experiment 3, we examined the generalizability of the effect of the diminutive form on the perceptual representation of objects.

## Experiment 1: Receiving a Gift

Receiving an unexpected gift should quite naturally improve one’s mood (Bohner et al., 1992).Yet, its linguistic presentation form might directly impact the extent of this impact. If the diminutive form is taken as a cue for physical size, we expect recipients’ mood to be less positive, whereas if the diminutive form is taken as a clue for its affective value, we expect recipients’ mood to improve. Inherent to the former expectation, we were also interested in the perceptual representation recipients may build when the coin is described with the diminutive form. Therefore, in Experiment 1, we examined whether the linguistic form of the object’s name given as a gift – a 1 Polish złoty coin worth approximately 32 cents – would lead to different satisfaction levels in the recipient as well as different perceptual representations.

### Method

#### Participants

Forty undergraduate students at the library of the University of Gdansk, Poland (25 women; *M*_age_ = 22.6, *SD* = 2.6) took part in this experiment. The experiment was approved by the university’s ethics committee, and all participants had granted their written informed consent after the experiment.

#### Design and Procedure

In this experiment, there were two conditions associated with the form in which the Polish złoty coin was presented: Diminutive vs. Regular form. Participants’ mood was measured 3 min after receiving the gift, as was done in Study 1A by Wilson et al. (2005).

During the experiment, an experimenter approached participants one at a time in the university library. Participants were initially given a card on which a Polish złoty coin was attached, with a simple “*Hi! This is for you, have a nice day*” greeting. After handing a card to a participant, the experimenter would walk away, as if they were trying to find somebody else to whom they could give the next card. Participants were randomly assigned to receive the złoty coin in one of two experimental conditions: a coin was attached to a paper card describing it in the diminutive (“*złotóweczka*” – small coin) or in its regular form (“*złotówka*” – a regular coin). We decided, as for all the experiments presented in this paper, to have Form (*diminutive* vs. *regular*) as a between factor, to avoid having any potential effect due to some pragmatic process (i.e., “*If I am given something in the diminutive form now, it is because I can assume that it is smaller*”).

Each version of the card was identical (726 mm × 127 mm, printed in color on a paper weighing 170 g/m2). At the top of the card it said “*This złotóweczka/złotówka is for you!*” and underneath, as in Wilson et al’s. (2005) procedure, it stated “*Who are we? A student society*” and “*Why do we do this? We promote random acts of kindness.*” In the upper right-hand corner, there was a yellow smiley face, and at the bottom of card, the phrase “*Have a nice day*” was placed.

Meanwhile, a second experimenter (unaware of the experimental conditions the participants were in) observed the scene from a distance, and, exactly 3 min after the first experimenter handed the card to a participant, they approached them and asked if the participants could fill out a questionnaire for their class project. The first part of the questionnaire consisted of some demographic questions, a general question about current positive and negative mood (“*How POSITIVE or NEGATIVE is your mood right now?*”), and six affective labels (joyful, pleased, frustrated, confused, alert, and agitated). Students answered questions about their mood on a 9-point Likert scale (*1 = very negative* to *9 = very positive*), and on the six affective labels [*1 = don’t feel (label) at all* to *9 = feel (label) a lot*]. The second part of the questionnaire focused directly on our experimental manipulation. The experimenter therefore told the participants that the next set of questions were directly related to them receiving the coin. Whilst he masked the card, he asked each participant to complete the questionnaire, which was composed of several questions assessing the participants’ attention to the card (e.g., “*Have you read the card carefully?*”, “*Since receiving the card, to what extent have you wondered why you received it?*”) and questions assessing the experimental manipulation *per se* (e.g., *What was the linguistic form used?*). Participants answered the *attention* questions on a 9-point Likert scale (*1 = not at all* to *9 = very carefully*). At the very end of the session, participants were asked to draw the size of the coin (without looking at it) and were asked if they had any clue about what hypothesis was being tested in this experiment (i.e., “*What do you think was the real purpose of this study?*”).

### Results and Discussion

All affective measures were assessed separately, and comparisons between the two experimental conditions were made by conducting independent *t*-tests when the sample distributions were normal, and Mann–Whitney tests when the sample distributions were not normal.

**FIGURE 1:**
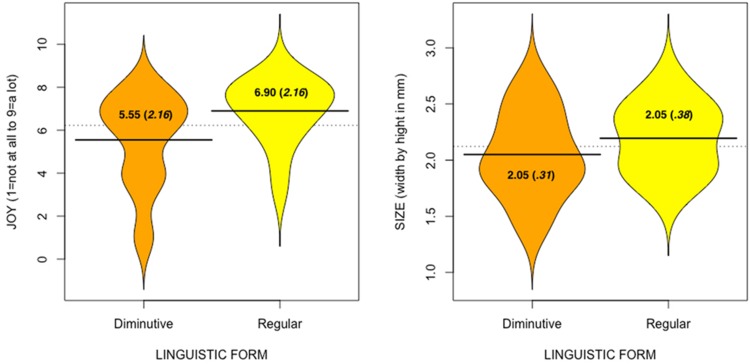
**Beanplots^1^ (with means, and standard deviations in brackets) of *joy* and coin size as a function of the linguistic form the coins were presented in**.

Out of the six affective scales, *joy*, as illustrated by the beanplots in **Figure 1**, showed a significant difference between those who received the złoty coin presented in its diminutive form (*M*= 5.55, *SD* = 2.16, *CI* [4.54–6.56]) and those who received the coin in its regular form (*M*= 6.90, *SD* = 1.52, *CI* [6.19–7.61], *U*(38) = 120.50, *Z* = -2.21, *p* = 0.03).

Participants who received the złoty coin presented in its diminutive form, as shown in the beanplots in **Figure 1**, on average, drew the coin as smaller than its actual (2.3 cm in diameter) size (*M*= 2.05 cm, *SD* = 0.38, *CI* [-0.43 – -0.07]), but it was only numerically different from the złoty coin presented in its regular form (*M* = 2.19 cm, *SD* = 0.31, *CI* [-0.25 – -0.04], *t*(38) = 1.32; *p*= 0.19). When directly comparing the two conditions, the difference did not reach significance. In fact, participants who received a coin described in the regular form also drew it smaller than the actual coin (yet not *as* small). In itself, this is not surprising, as people are quite bad at reproducing items, even when they have often been exposed to them (e.g., Blake et al., 2015).

Participants who received the złoty coin presented in its diminutive form also declared having read the card more carefully (*M* = 8.30, *SD* = 0.92, *CI* [7.86 – 8.73]) than participants who received the złoty coin presented in its regular form (*M* = 7.25, *SD* = 1.48, *CI* [6.55 – 7.94], *U*(38) = 117.00, Z = -2.26, *p* = 0.02). This effect could illustrate the fact that participants noticed the diminutive form, yet they failed to uncover the purpose of the experiment, as indicated by the responses to the final question.

This first experiment suggests that the linguistic form of an object had an impact on the related mood of the participants. This effect is quite fascinating, inasmuch as the coin received is well known and frequently used. People are exposed to it on a daily basis. However, since its actual value is set, we cannot truly extend our findings to saying that the diminutive form also has an effect on the perceived value of the object. We therefore conducted Experiment 2 to examine this idea.

## Experiment 2: Value of a Product

The aim of Experiment 2 was to determine if the use of a diminutive form of an object could affect how people evaluate its quality and pecuniary value. If the effect found in Experiment 1 held true even for an (supposedly) objective evaluation, it would provide us with strong support that linguistic forms have considerable implications as to the way we mentally represent objects.

In this experiment, to avoid fixed values (i.e., a złoty coin is always worth the same amount) we used a more applied setting: an online auction. This choice is also supported by the fact that, at Polish auctions, sellers frequently use diminutive forms in the descriptions of their objects, even if the objects are clearly full-sized (e.g., big SUV cars presented as *autko – a car*_with diminutive_). This suggests that some sellers strategically want to highlight the emotional value of their objects (i.e., “*it used to be my favorite car*”) whilst underestimating the meaning of smallness they might convey. In essence, in this experiment, we investigated whether this is a good strategy or not.

### Method

#### Participants

Ninety-eight participants (48 women; *M*_age_ = 24.70, *SD*= 5.37) recruited online via an educational website took part in this experiment, in exchange for being entered in a raﬄe to win educational books. The experiment was approved by the university’s ethics committee, and all participants had granted their written informed consent before starting the experiment.

#### Design and Procedure

As in Experiment 1, there were two conditions associated with the form in which the items were presented: Diminutive vs. Regular form, and participants rated their mood after having received a złoty coin. In this experiment, participants’ judgments of the monetary value, as well as their evaluation of the pieces of clothing for auction were measured.

Six pictures of new pieces of clothing (i.e., a man’s coat, a woman’s sweater, a man’s tracksuit, women’s shoes, men’s sunglasses and a woman’s jacket) were selected for the experiment. All items were presented as if they were for sale on a simulated website that mirrored the most popular Polish online auction website (*allegro.pl*, which operates similarly to *ebay.com*). In actual fact, all six items had been for sale before, at the exact price of 150 PLN. All pictures were digitally altered to hide any visible trademarks. All item descriptions were matched to each other in terms of word count and number of characteristic features. Before the actual evaluation of the items, participants’ mood was measured using two positive and two negative mood items (Wojciszke and Baryła, 2005). This was done to ensure that any potential evaluation variance was attributable to the use of the diminutive form and not to a possible mood effect (i.e., merely *seeing* an object written in the diminutive form alters one’s mood).

Forty-seven randomly chosen participants were redirected to a questionnaire describing the items of clothing in a diminutive form (eg. *płaszcz-yk* (coat_with diminutive_); *dres-ik* (tracksuit_with diminutive_), and the remaining 51 participants were redirected to a questionnaire describing the items of clothing in a regular form (e.g., *płaszcz, dres*). Participants read the item descriptions, inspected the accompanying pictures, and answered three questions concerning each item. Two questions pertained to the value of the presented item of clothing (“*What do you think the ‘buy now’ price would be”?* and*“What is the minimum price the seller would be willing to accept for this item?”*; both answers were given in Polish złoty) and a single question addressed the credibility of the auction itself (“*Does the actual condition of the clothing match the provided description?”*), evaluated on a 7-point Likert scale (*1 = absolutely not credible* to *7 = absolutely credible*). After participants judged all six of the products, their mood was evaluated again and we probed them for suspicions about our hypothesis. Three of the participants presented with descriptions in the diminutive form noticed that the clothing names were presented in a diminutive form, yet they displayed no suspicion about the nature of our study. They were therefore included in the analyses.

### Results and Discussion

Judgments across all six objects were collapsed into indices of both price judgments and auction credibility (*Buy now price*: *Cronbachs’*α = 0.83; *Minimum price*: α = 0.77; *Credibility*: α = 0.66). As in Experiment 1, we compared the two experimental conditions for each measurement (i.e., each dependent variable) by conducting independent *t*-tests when the sample distributions were normal, and Mann–Whitney tests when the sample distributions were not normal.

#### Buy Now Price

**FIGURE 2:**
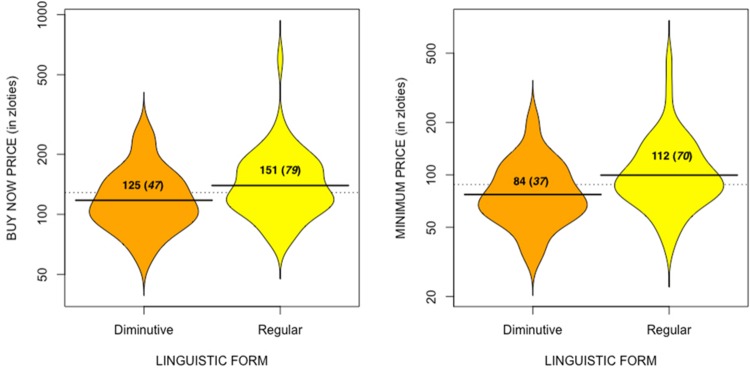
**Beanplots (with means, and standard deviations in brackets) of *buy now price* and *minimum price* as a function of the linguistic form the coins were presented in**.

As predicted, and as illustrated by the beanplots in **Figure 2**, there was a main effect of the linguistic form used, U(96) = 869.50, Z = -2.34, *p* = 0.02; indicating that participants who read the diminutive form descriptions of the items attributed less value to them (*M* = 124.82; *SD* = 46.49, *CI* [111.17–138.47]) than those who read the regular form descriptions (*M* = 151.24; *SD* = 78.54, *CI* [129.15 – 173.33]). In a similar way as for the złoty coin size in Experiment 1, given that we knew the actual price paid for the depicted items of clothing, we also ran a one-sample Wilcoxon sign test comparing the means for both groups to the fixed value (150 złoties). Participants who read the diminutive form descriptions were significantly (*p*< 0.001) less accurate in their judgments of the *buy now* price, whilst those who read the regular form descriptions estimated the price more in accordance with their real market value.

#### Minimum Price

As seen in **Figure 2**, the same pattern of results emerged from the participants’ judgments of the items’ *minimum* price: a predicted main effect of the linguistic form used, *U*(96) = 863.00, *Z* = -2.81, *p* < 0.01, meaning that participants who read the diminutive form descriptions indicated lower prices (*M* = 83.82 Polish złoty; *SD* = 37.30, *CI* [72.86–94.77]) than those who read the regular form descriptions (*M* = 112.40 Polish złoty; *SD* = 69.70, *CI* [92.79– 131.99]).

#### Auction’s Credibility and Participants’ Mood

There was no significant difference as to the items’ credibility [*U*(96) = 1123.00, *Z* = -0.538, *ns*] nor was there a difference in terms of mood [*U*(96) = 1171.00, *Z* = -0.193, *ns*], suggesting that the effects found were entirely attributable to the linguistic forms used, and to their impact upon quality and monetary value attributions.

In all, we found that the diminutive form influenced the participants’ evaluations of the objects to be sold. Taken together with the results from Experiment 1, using diminutive forms (instead of the regular form) to describe an object led to less satisfaction when receiving the object as a gift (Experiment 1), and to a smaller monetary value when it was to be sold on an online auction website (Experiment 2).

Inherent to both Experiments 1 and 2, however, is the assumption that the diminutive form, as mentioned in the introduction, is associated to the mental processing of size. Although we do believe this to be true, we have yet to directly show the direct effect of the diminutive on *perceptual representations* (other than the coin measurement in Experiment 1). In addition to this, although there was a variety of stimuli in the two experiments reported so far, the question of generalizability remains. In both experiments, only by-participant analyses were performed, leaving by-item generalizability to assumptions. In this final experiment, we directly tested the effect of the diminutive form on perceptual representations by asking participants to *select* a sample of items (fruits and vegetables), either written in the regular or the diminutive form, from a set of size varying choices.

## Experiment 3: Selecting from Fruits and Vegetables of Different Sizes

The purpose of our final experiment was threefold. First, we wanted to make sure that the effects we obtained in the previous experiments were driven by perceptual representations. Since we did not directly test this (except from the drawings in Experiment 1), those effects obtained in all experiments could be attributed to alternative processes. For example, one could argue that objects in the diminutive form may simply be considered as “more negative,” hence dissociated from any perceptual representation. The consequent effects would be similar to those observed. We therefore decided to manipulate the size of the items (i.e., fruits and vegetables) presented to participants and engage them in a direct and explicit *exemplar comparison* and *selection* task, whereby we hypothesized that participants’ comparisons and selections would be dependent upon the grammatical form the items were presented in. In this experiment, we therefore presented participants with items of different sizes, and asked them to carefully inspect them and select a sample of them. Second, we wanted to examine the item generalizability of the perceptual effects of the diminutive form. We therefore increased our item sample (*N* = 20) to include a wide variety of fruits and vegetables and ran both by-participant and by-item analyses. Last but not least, we wanted to keep the task at hand as ecological as possible. We therefore constructed a *grocery task*, in which participants had to pick some fruits and vegetables, as if they were participating in a grocery shopping assessment survey. By having this ecological cover story, we ensured that our manipulation was hidden from the participants.

### Method

#### Participants

Four hundred and six participants (343 women; *M*_age_ = 23.2, *SD* = 7,5) recruited online via an educational website took part in this experiment, in exchange for being entered in a raﬄe to win educational books. One female participant had to be removed from the analyses due to incomplete data. The experiment was approved by the university’s ethics committee, and all participants had granted their written informed consent before starting the experiment.

#### Materials

**FIGURE 3:**
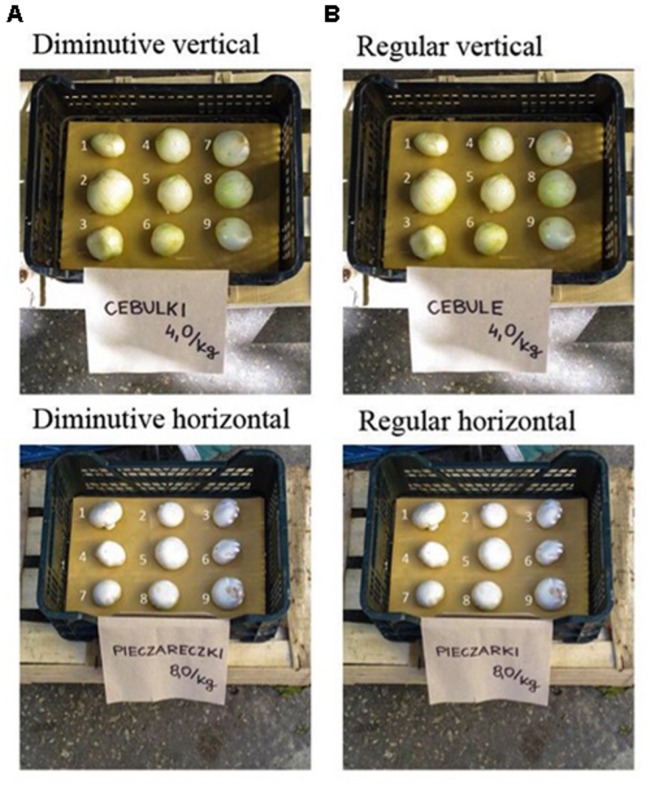
**Examples of stimulus used in Experiment 3. (A)** Presents objects in diminutive form while **(B)** presents the regular form of the label. Also, two methods of numbering the objects (counterbalanced between participants) were used: Top Panel presents vertical numbering, while Bottom Panel presents horizontal numbering of fruits and vegetables.

In this experiment, we presented participants with 20 box containers, each comprising nine exemplars, varying in size, of a fruit or a vegetable^2^ (e.g., one box had nine white onions). As explained in detail later, participants, for each fruit or vegetable, had to pick three exemplars. In this experiment, and mimicking Experiments 1 and 2, we wanted to make sure that our task of selecting fruits or vegetables was as ecological as possible (see **Figure 3**, for an example of a box containing mushrooms and lemons). There were two important methodological consequences to this drive for ecological validity. First, we decided to use one (picture of a) single box container (size: 60 cm × 40 cm × 22 cm) in which to present the objects, to mirror product presentation at fruit and vegetable markets. By doing so, we generated a very salient cover story, inducing participants into thinking that they were participating in a grocery shopping assessment survey (i.e., distracting from any explicit notice of the use, in one task, of the diminutive form). Second, we presented fruits and vegetables that were reasonable in size and perceptually realistic. Consequently, there were, naturally, smaller (i.e., white onions) as well as bigger ones (e.g., celeries). As the differences in sizes within the nine exemplars of each fruit or vegetable was constant across types of fruits or vegetables, we expected size differences between exemplars of bigger fruits or vegetables to be more difficult to perceive than those of smaller fruits or vegetables. For smaller fruits or vegetables, a bigger proportion of the actual size changed between the exemplars. Since, we were expecting that the diminutive form could affect the perceptual representations of the fruits and vegetables, we separated our items into *smaller* and *bigger* fruits or vegetables in all analyses, expecting our effects to be smaller for the bigger fruits or vegetables, as the relative size difference is less perceptually profound. Note that there were no differences between fruits and vegetables, hence we did not treat item *nature* (i.e., fruit vs. vegetable) as a factor.

#### Procedure

To encourage participants to believe that the whole task was about their grocery shopping habits, we first asked participants two questions pertaining to these habits (i.e., “*How often do you shop”* and “*For how many persons do you shop”*). After having responded to these questions and to ensure that participants would notice the labels (i.e., for some participants with the diminutive form) and read them carefully (ensuring the activation of any processes associated to language form), we had an initial sorting task in which participants had to simply match six randomly chosen fruit or vegetable labels with their corresponding pictures. The labels were written in the same way as for the experimental task. Although this pre-task was aimed at ensuring proper attention to the labels, it also further supported our cover story.

In the experimental task, participants were then randomly presented with a series of 20 boxes, one at a time, each containing nine fruits or vegetables^3^. At the bottom of each box, the name of the fruit or the vegetable, in the diminutive (for 196 participants) or the regular form (for 210 participants), was written, along with a price (constant across language form), as is habitually done at grocery markets (**Figure 3**). Participants were simply asked to *choose three exemplars* as they would do when shopping for groceries. Each fruit in the box was marked with a number (also counterbalancing between two methods of numbering objects), and participants had to simply type three numbers in and proceed to the next fruit or vegetable. At the end of the task, we asked participants to type in what the experiment was about. None of the participants guessed the right hypothesis.

### Results

#### Data Preparation

When proceeding with the analyses, we realized that there was one perceptual issue that we did not foresee. If, as we expected, participants make judgments based on the perceptual size properties of the fruits and vegetables, we cannot be sure as to the actual perceptual base for making these judgments. In other terms, we cannot be sure that participants use the actual size, in terms of surface (as we measured in pixel square), in terms of width, or in terms of height of the fruits and vegetables. In fact, participants’ perceptual frame of reference may vary from one vegetable (e.g., using the width for a carrot sitting horizontally) to another (e.g., using the height for an onion). To avoid making such a decision ourselves and using a perceptual frame that might not be correct for a particular fruit or vegetable, we conducted an extra experiment to determine an appropriate measure of perceptual size.

**FIGURE 4:**
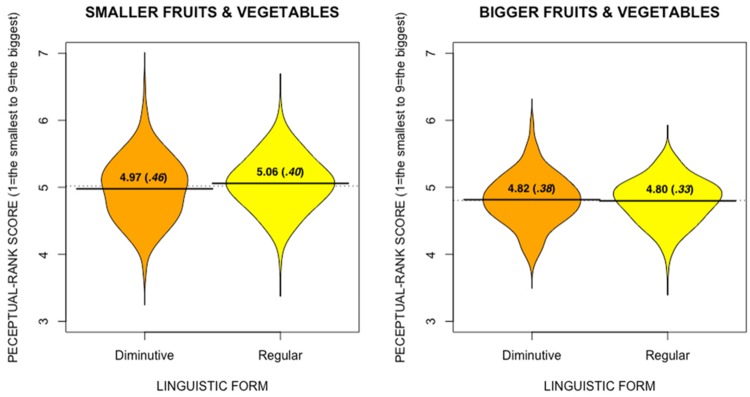
**Beanplots (with means and standard deviations in brackets) of the mean perceptual-rank scores of the three chosen fruits or vegetables in Experiment 3, according to Actual Size and Language Form**.

In this extra experiment, we asked 53 participants, via an online questionnaire, to rank, for each fruit and vegetable that we used in the experiment, the nine related exemplars from the *smallest* to the *biggest* one. As the task was quite resourceful and time consuming, we asked the participants to only evaluate 10 randomly selected fruits or vegetables out of the 20 (yet counterbalanced so that we had all fruits or vegetables ranked at least 26 times). After having ranked the nine exemplars of a fruit or vegetable, participants had to evaluate (using a slider anchored from 1 = *very easy* to 21 = *very difficult*), how difficult the task was, as well as how sure they were of their ranking (from 1 = *not sure* to 21 = *very sure*). Importantly, and confirming our expectations regarding bigger and smaller fruits or vegetables, bigger fruits or vegetables were more difficult to rank, *t*(18) = 2.64, *p* < 0.05^4^ (smaller fruits or vegetables: *M*= 11.80, *SD*= 1.88; bigger ones: *M*= 9.48, *SD*= 2.05). For the purpose of subsequent analyses, we calculated, for each single exemplar of a fruit or vegetable, its mean position within the nine related exemplars (i.e., one being the smallest, and nine being the biggest). For example, one particular cucumber would have a mean of 2.2, suggesting that it was ranked mostly at the second position (i.e., the second smallest cucumber). Our dependent variable was henceforth this perceptual-rank score (**Figure 4**), and we used this perceptual rank-score as the dependent variable in all subsequent analyses.

#### Analyses

To examine whether the diminutive form had an impact on the way participants represented the fruits and vegetables, henceforth affecting the selection of the three exemplars, we conducted a 2 (Actual Size: Smaller vs. Bigger) × 2 (Language: Regular vs. Diminutive) mixed ANOVA, with Actual Size as a within-participant factor and Language as a between-participant factor in the by-participant analyses, and Actual Size as a between-item factor and Language as a within-item factor in the by-item analyses. In this experiment, all sample distributions were normal.

Overall, there was only one effect that reached significance both by participants and by items: An Actual Size by Language interaction, *F*_1_(1,403) = 4.62, *p*= 0.03, *F*_2_(1,18) = 8.92, *p*< 0.01. As shown in **Figure 2**, the effect of language form was only apparent in smaller fruits or vegetables (i.e., those easier to perceive in terms of size changes) [smaller fruits or vegetables: *t*_1_(403) = 2.04; *p*< 0.05, *t*_2_(9) = 3.17; *p*= 0.04 with Bonneferoni correction; bigger fruits or vegetables: *t*_1_(403) = -0.50; ns, *t*_2_(9) = -0.77; *ns*] ^5^.

### Discussion

In this experiment, we directly tested the perceptual representations generated by the use of the diminutive form by asking participants to *select* a sample of fruits or vegetables. As expected, when written in the diminutive form, participants did select smaller exemplars. Importantly, we decided to mask the purpose of the experiment from the participants by convincing them that they were participating in a *realistic* grocery shopping assessment survey. One of our primary purposes was to examine the item generalizability (and not only participant generalizability as in Experiments 1 and 2) of the perceptual representation effects due to the use of diminutive forms, and although we showed that this effect was generalizable, it is only true when fruit or vegetable size differences are perceptually easy to recognize. More specifically, in our experiment, to keep a realistic and ecologically valid manipulation, we decided to manipulate size by constant differences (in mm^2^) across all fruits or vegetables. Had we manipulated size by targeting proportional differences (i.e., a certain proportion of any fruit), we would have had similar effects across *all* fruits or vegetables. Yet, considering the constraining factor of the box container – and to keep it realistic –, this was not possible. As a matter of fact, in retrospect, we do not feel that this is a problem, inasmuch as this issue even substantiates our argument that the diminutive form is associated to perceptual representations: when it is difficult to perceive size differences between a bigger and a smaller item, the effect of the diminutive form is absent. Note that although we cannot truly rule out the idea that the diminutive form may trigger an effect of valence (i.e., diminutive = negative), it is now hard to deny that the diminutive form *is* associated to perceptual representations (including encoding, memory,…).

## General Discussion

Across three experiments, we demonstrated that people mentally process objects differently when these objects are presented in a diminutive form than when they are presented in the regular form. That is, although the physical properties of the objects remained constant across conditions in Experiments 1 and 2, the objects were mentally processed differently depending on the linguistic form (i.e., with or without the diminutive form) in which they were presented. People were less satisfied with having received the objects (Experiment 1) and mentally processed the objects as less valuable (Experiment 2). When the objects (here fruits or vegetables) varied in size, smaller ones were more often selected when described in a diminutive form (Experiment 3). In Experiment 1, participants’ mood after receiving a gift was lower when the gift was presented in the diminutive form than when presented in the regular form. In Experiment 2, various pieces of clothing were valued as being cheaper when they were presented in the diminutive form than when presented in the regular form. Finally, the results of Experiment 3 demonstrate that the effects under investigation are truly associated to size representation and are generalizable not only across participants, but also across items.

Note that the reported perceptual effects of diminutives are reminiscent of *El Greco’s fallacy* (Firestone and Scholl, 2014). The *El Greco fallacy* refers to a false interpretation of the artworks of a famous painter of the Spanish Renaissance, who had a signature technique in reproducing human portraits as being oddly elongated. Art historians in the early 1900s suggested that his technique must have been the result of his strongely distorted vision. It was assumed that he must have been a suferer of severe astigmatism and he was merely painting exactly how he saw the world. In fact this cannot possibly be true, but it took almost a century to falsify this interpretatation. Had this interpretation been true, El Greco would have seen his canvases distorted in the same way (i.e., elongated canvases), and painting onto them would have canceled out any true visual distortion. Thus, as mentioned in the introduction, we do not believe that the diminutive form directly affects perception, or vision, as the results of Experiment 3 would be very different if this was the case. That is, if the diminutive form resulted in (all) the objects being *seen* as smaller, participants would have picked *actual* bigger fruits and vegetables (i.e., not noticing that they were the big ones).

Still, altogether, we showed that (a) the diminutive form is spontaneously processed as illustrative of an object size, and (b) that its resulting represented size is used as a heuristic cue on which to base different evaluative processes. Consequently, our results raise several issues. First, they demonstrate that subtle language differences can have a significant impact upon the perceived properties of objects, supporting Slobin’s (2003) idea that language shapes our representations. Although very little research has been conducted on the impact of diminutive forms upon mental representations, our results clearly complement previous research on the more general influence of language on cognitive processes (Loftus and Palmer, 1974; Maass and Russo, 2003; Winawer et al., 2007; Casasanto and Boroditsky, 2008; Fiedler, 2008; Boroditsky, 2011; Lupyan, 2012).

Similarly to the use of diminutive forms to refer to objects, these forms can ambiguously refer to size as well as to affection (Haman, 2003; Kray et al., 2012). Since physical size has actually been shown to be considered as a cue for social status (Campbell, 1976; Schubert et al., 2013), referring to human beings using diminutive forms could generate conflicting representations, which in turn, could be grounded on social dimensions (such as sexism, for example). Future studies should address the hypothesis of examining the influence of diminutive forms when addressing other people (Merkel et al., 2012; Formanowicz et al., 2013).

Second, our results also support the idea that, for several evaluative dimensions, size does matter. Although it might no longer be the case, some authors (e.g., Leaky, 1971) have argued that, in evolutionary terms, the size of objects may have been a very useful heuristic in preference judgments. The heuristic “bigger is better,” which we believe is at the heart of the effects in the first two experiments, may still be used as a cue for preference. Some would argue that, at present, many objects of smaller size are commonly preferred (e.g., computers, phones or microchips), suggesting that the heuristic “smaller is better” can also be learnt (Silvera et al., 2002). Future studies should address this. For example, activating the “smaller is better” heuristic as the point of reference may actually reverse the pattern of results found in the present series of experiments.

## Conclusion

The current research emphasizes the importance of language forms when describing objects. The names of objects described using the diminutive form are interpreted very differently than when described in the regular form. This is true in terms of the resulting mood when receiving an object as much as the quality evaluation of the objects. Since size *does* seem to matter, the use of diminutive forms can truly undermine the qualitative value of objects.

## Author Contributions

MP initiated the project with KB. All experiment were ran in Poland. PG and MP designed Experiment 3. All three authors actively participated in the writing up of the paper.

## Conflict of Interest Statement

The authors declare that the research was conducted in the absence of any commercial or financial relationships that could be construed as a potential conflict of interest.

## Footnotes

1 A beanplot is a combination between a scatter plot and a density distribution trace (for details, see Kampstra, 2008). In a nutshell, a density function (Kernel density estimation in our case) describes the relative likelihood of certain values. As such, they provide us with an estimation of the distribution likelihood that any sample of the drawn population would take. The beanplots were generated using the R software (R Core Team, 2013) and the *beanplot* function from the *beanplot* package (Kampstra, 2008).

2 The 20 objects we used in Experiment 3 were: apricots (morelki vs. morele), beetroots (buraczki vs. buraki), carrots (marchewki vs. marchew), lemons (cytrynki vs. cytryny), green apples (zielone jabłuszka vs. jabłka), red apples (czerwone jabluszka vs. jabłka), gurkens (ogóreczki vs. ogórki), yellow peppers (żółte papryczki vs. papryki), red peppers (czerwone papryczki vs. papryki), oranges (pomarañczki vs. pomarañcze), long tomatoes (podłużne pomidorki vs. pomidory), round tomatoes (okraaaagłe pomidorki vs. pomidory), yellow onions (żółte cebulki vs. cebule), white onions (białe cebulki vs. cebule), peaches (brzoskwinki vs. brzoskwinie), pears (gruszeczki vs. gruszki), yellow plums (żółte śliweczki vs. śliwki), violet plums (fioletowe śliweczki vs. śliwki), celery (selerki vs. selery), and mushrooms (pieczareczki vs. pieczarki).

3 To decide on the actual size of each exemplar of the 20 fruits or vegetables, we calculated the *actual surface* (in pixels) of each fruit or vegetable using ImageJ 1.48v Software. Variations between fruits or vegetables within a box of nine exemplars were on average of 994 pixel square (approximately 69 mm^2^).

4 Quite logically, the easier a fruit or vegetable was to rank, the more certain the participants were to be right, *r*(20) = 0.96, *p*< 0.001.

5 There was also a main effect of Actual Size, yet only significant when participants were considered as a random effect, but not when items were, *F*_1_(1,403) = 73.42, *p*< 0.05, *F*_2_(1,18) = 4.24, *p* < 0.06. Note that this effect is interesting in itself, as it suggests that when fruits or vegetables have to be chosen from *bigger ones*, participants tend to go for the slightly smaller ones.
